# Impact of pulmonary vein anatomy and ostial dimensions on long-term outcome after single-shot device–guided cryoablation for paroxysmal atrial fibrillation

**DOI:** 10.1007/s10840-023-01554-4

**Published:** 2023-05-05

**Authors:** Khuraman Isgandarova, Leonard Bergau, Mustapha El Hamriti, Martin Braun, Misagh Piran, Guram Imnadze, Moneeb Khalaph, Stephan Molatta, Vanessa Sciacca, Thomas Fink, Philipp Sommer, Denise Guckel, Christian Sohns

**Affiliations:** 1 Clinic for Electrophysiology, Herz- und Diabeteszentrum NRW, Ruhr-Universität Bochum, 32545 Bad Oeynhausen, Germany; 2 Institute for Radiology, Nuclear Medicine and Molecular Imaging, Herz- und Diabeteszentrum NRW, Ruhr-Universität Bochum, 32545 Bad Oeynhausen, Germany

**Keywords:** Atrial fibrillation, Cryoballoon, Magnetic resonance imaging, Pulmonary vein anatomy, AF-recurrence

## Abstract

**Background:**

Cryoballoon (CB)-guided pulmonary vein isolation (PVI) is an established treatment for atrial fibrillation (AF). This observational study aimed to assess the role of individual anatomical characteristics to predict long-term freedom from arrhythmia recurrence after CB-guided PVI for paroxysmal AF (PAF).

**Methods:**

Three hundred fifty three consecutive patients (58 ± 11 years, 56% males), undergoing PVI between 2012 and 2018 were analysed. Individual pulmonary vein (PV) anatomy was assessed using preprocedural cardiac magnetic resonance imaging (MRI). For each PV, the cross-sectional area (CSA) was calculated. The impact of PV characteristics and CSA on long-term AF-free survival was evaluated.

**Results:**

Acute PVI was achieved in all patients. Two hundred twenty-three patients (63%) had a normal PV anatomy (2 left- and 2 right-sided PV). Variant PV anatomy was present in 130 patients (37%). During the observation period of 48 months, AF-recurrence was documented in 167 patients (47 %). Patients with AF-recurrence presented with significantly enlarged right-sided PVs and left superior PVs (LSPVs) (*p* < 0.001). The presence of left common PVs (LCPVs) (*n* = 75, Log-rank *p* < 0.001) as well as right variant PVs (*n* = 35, Log rank *p* < 0.001) was associated with a significantly impaired long-term AF-free survival rate as compared to patients with normal PV characteristics.

**Conclusion:**

Variant PV anatomy is a good predictor for AF-recurrence. A correlation between an enlarged CSA of right-sided PVs as well as LSPVs and AF-recurrence was documented.

## Introduction

Atrial fibrillation (AF) is the most common rhythm disorder, which is often associated with an impaired quality of life, stroke, dementia, and heart failure [1, 2]. Interventional therapy has become a class IA-recommendation for symptomatic drug-refractory paroxysmal AF (PAF) [1]. The FIRE-ICE study identified cryoballoon-ablation (CBA) as non-inferior to radiofrequency ablation (RFA) regarding efficacy and safety aspects [2]. Compared to RFA, CBA is associated with a rapid learning curve [3] and shorter procedure duration [2]. CBA results in a favourable long-term outcome [4]. Meanwhile, novel balloon-guided single-shot devices have been introduced [5]. Nevertheless, PV-reconnection remains an issue [5, 6]. Since complete occlusion of the PV ostium is the cornerstone for PVI, individual anatomical characteristics and ostial dimensions of PVs may have an impact on AF-free survival. In this context, long-term follow-up data are lacking.

## Methods

### Patients

This observational single-centre study included 353 consecutive patients undergoing CB-guided catheter ablation for symptomatic and drug refractory PAF between 2012 and 2018. Patients with a left ventricular dysfunction, a relevant mitral valve disease and a previous AF ablation were excluded. Patients were divided into two groups: regular versus variant pulmonary vein anatomy. We compared clinical characteristics and effects of PV anatomy on freedom from arrhythmia recurrence. The diagnosis of PAF was made using the definition from the ESC guidelines for the diagnosis and the management of AF [1]. Arrhythmia recurrence was defined as documented episode of any atrial tachycardia (AT)/AF > 30 s. AF-recurrence within the first 12 months excluding the blanking period of 90 days is defined as “early AF-recurrence.”

### Procedural management

All patients underwent preprocedural transoesophageal echocardiography to rule out left atrial thrombus formation. Antiarrhythmic agents (AADs) except for amiodarone were discontinued at least 48 h before ablation. Anticoagulation with phenprocoumon was continued aiming for an International Normalized Ratio (INR) between 2.0 and 3.0. Direct oral anticoagulants (DOAC) were stopped one half-life before ablation. Pericardial effusion was ruled out immediately after ablation and the next day thereafter. Anticoagulation was continued within 4 h after the procedure with phenprocoumon or DOAC when there was no evidence for pericardial effusion. AADs were prescribed to the operators’ discretion for a period of 3 months following ablation.

### Assessment of PV anatomy and size

**Fig. 1:**
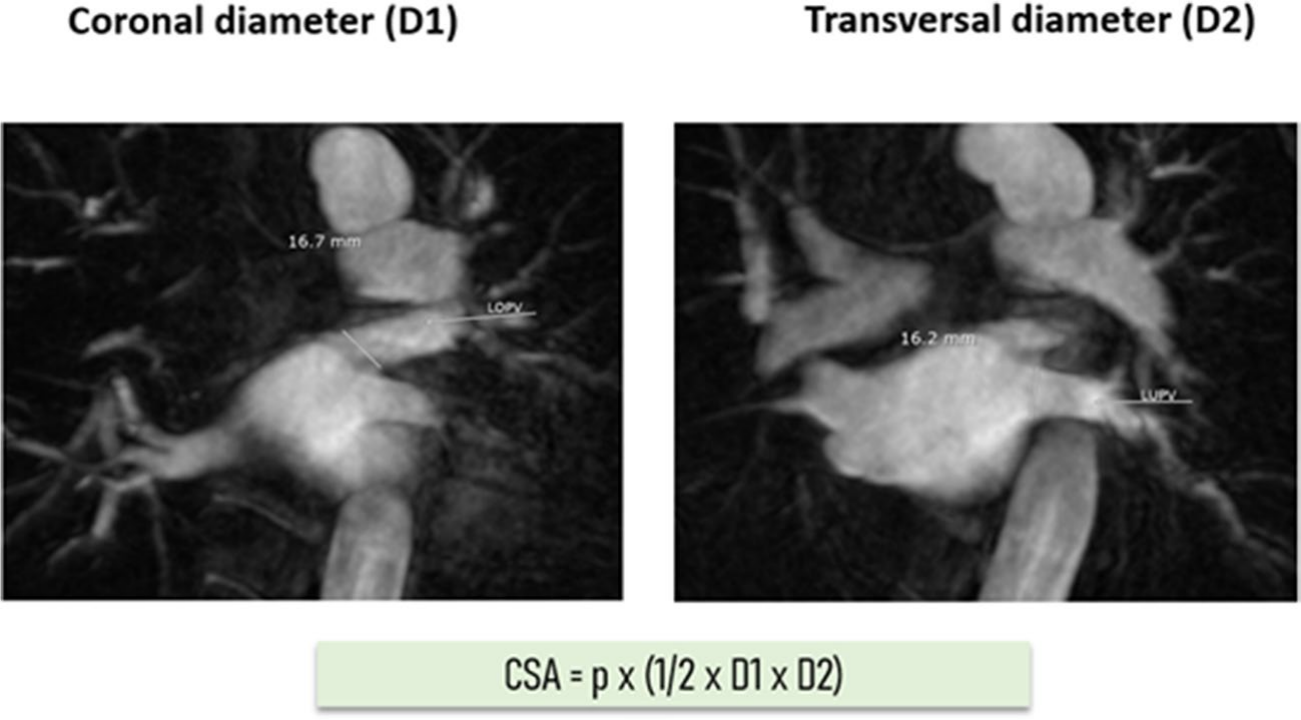
MRI-based measurement of the PVs’ coronal (D1) and transversal diameter (D2). MRI, magnet-resonance imaging; PV, pulmonary vein; CSA, cross-sectional area; D1, coronal diameter; D2, transversal diameter; *p*, Pi-number, 3.14; LOPV, left superior pulmonary vein; LUPV, left inferior pulmonary vein

All patients underwent MRI with (1.5 or 3 Tesla) prior to the ablation procedure. Cine imaging was performed in an end-expiration breath-hold using a standard multislice balanced steady-state free precession (bSSFP) technique (effective TR 2.7 ms, TE 1.3 ms, 1.4 × 1.4 mm^2^ in-plane, slice thickness 10 mm, 50 phases). Contrast was injected with a dose of 0.1–0.2 mmol/kg at a rate of 1–2 mL/s, followed by saline flash. CSA was determined according to the established method described by Kato et al. In detail, PV diameters were measured in superoinferior and anteroposterior directions. To determine the long axis of the PV, axial and coronal tomograms at the junction of the PV and LA were displayed, and then oblique coronal and oblique axial images were identified parallel to each PV. The PV ostium was defined as the point of inflection between the LA wall and the PV wall [7]. Along with conventional MRI scans, we additionally reconstructed a 3D model of each PV, which facilitated the distinction between ostium and antrum. The size of each PV was given in coronary (D1) and transversal (D2) diameter. The cross-sectional area (CSA) was calculated using the following equation, *p* × (1/2 × D1 × D2) allowing for an approximate determination of the PV ostium dimension (Fig. 1).

### Ablation procedure

The procedure was performed under conscious sedation with propofol and sufentanil. Intravenous heparin was administered to maintain an activated clotting time (ACT) of 300 s throughout the procedure.

The 28-mm AFA cryoballoon (Arctic Front Advance, Medtronic) was applied in all procedures. Following transseptal puncture, the balloon device was advanced into the LA via a steerable sheath (15-F FlexCath advance Medtronic). A multipolar mapping catheter (Achieve Advance Mapping Catheter, Medtronic) was used for PV mapping.

Diaphragmatic excursion during ablation of the right-sided veins was confirmed via continuous stimulation of the phrenic nerve and compound motor action potential (CMAP) visualization. Complete PV-occlusion prior to each freezing cycle was confirmed by PV-angiography with contrast media injection with the balloon inflated and placed at the PV-ostia. If an antral LCPV occlusion could not be obtained, a sequential ablation approach was adapted. At first, the first superior branch of the LCPV was targeted, followed by ablation of the first inferior branch. To verify complete occlusion of the targeted LCPV branch, contrast medium was injected. To achieve complete occlusion of RMPVs, the ablation technique described above was applied. In addition, “hockey stick,” “loop,” and “pull-down” techniques were performed, if necessary. Ablation was performed adherent to a 2*180s freeze per vein protocol. Persistent PVI (entrance and exit block) was confirmed after a waiting period of 20 min. Bonus freezes were not applied during standard protocol ablation but could have been used at the discretion of the operator. If isolation was not achieved, touch-up ablation with a focal cryo-energy catheter (Freezor MAX; Medtronic) was performed.

### Follow-up

After discharge, follow-up visits were scheduled at 3, 6, and 12 months after the procedure, including 7-day-Holter-ECGs and interviews. Thereafter, 24-h-Holter ECGs were performed every 6 months during the whole observation period of 48 months. Additional unscheduled visits were performed in case of complaints. The total observation duration was 48 months. Most patients were followed up in our outpatient clinic. The other patients kindly provided their follow-up reports on our request.

### Statistical analysis

All calculations were performed using the statistical package for the social sciences (IBM SPSS Statistics 27.0). The chi-squared test was used for categorical variables and Student’s *t* test for comparison of continuous variables. Data are presented as mean ± standard deviation or percentage value (%) unless stated otherwise. A two-tailed *p*-value < 0.05 was considered statistically significant. Multivariate analysis was conducted using multiple logistic regression. Data processing and analysis was performed using Excel 2010 (Microsoft Corp., Redmond, WA, USA). Event-free survival was calculated by Kaplan–Meier analysis as time from initial PVI to first documented AF/AT episode > 30 s. AF-recurrence within the blanking period of 90 days was excluded from data analyses. The log-rank test was used to assess differences in event-free survival time between two groups: normal versus variant PV anatomy.

## Results

### Study population

The study population consisted of 353 consecutive patients (58 ± 11 years old, 56% males) who underwent an index CBA for symptomatic PAF. Baseline parameter is summarized in Table 1. No major, relevant differences were observed between the groups.

**Table 1:** Patients’ baseline parameter and procedural characteristics. Continuous variables are shown as the mean ± SD and categorical variables as the number (%). A *p*-value < 0.05 and bold letters state statistical significance. *BMI* body mass index, *DM* diabetes mellitus, *CAD* coronary artery disease, *OSA* obstructive sleep apnoea, *LA* left atrium, *AP-diameter* anterior-posterior diameter, *AADs* antiarrhythmic drugs

Parameter/pat. group	Study population (*n* = 353)	Patients with regular PV anatomy (*n* = 223, 63%)	Patients with variant PV anatomy (*n* = 130, 37%)	*p*-value	Long-term AF-free survival (*n* = 186, 53%)	Patients with AF-recurrence (*n* = 167, 47%)	*p*-value
Sex (male)	209 (59 %)	134 (60%)	75 (58%)	0.93	103 (55%)	93 (56%)	0.73
Age, years	58 ± 11	57 ± 7	56 ± 9	0.87	57 ± 12	56 ± 11	0.99
BMI, kg^2^/cm	27.8 ± 4	27.4 ± 4	26.8 ± 3	0.83	26.9 ± 4	27.4 ± 4	0.86
Smoking	63 (18 %)	41 (18 %)	22 (17%)	0.93	33	30	0.96
Arterial hypertension	198 (56 %)	127 (57 %)	75 (58 %)	0.96	102 (54 %)	96 (57 %)	0.42
DM	14 (4 %)	8 (4%)	6 (5%)	1.4	9 (5%)	5 (3 %)	0.11
CAD	23 (7 %)	14 (6%)	9 (7%)	1.6	11 (6%)	12 (7 %)	0.21
OSA	22 (6 %)	16 (7%)	6 (5%)	0.92	9 (5%)	13 (7 %)	0.48
CHA2DS2-VasC-Score	1.2	1.3	1.1	0.9	1.2	1.3	0.33
LA AP-diameter, mm	38 ± 7	37 ± 5	38 ± 7	0.91	38 ± 6	39 ± 5	0.32
AADs	92 (26%)	60 (27%)	32 (25%)	0.79	47 (24%)	45 (27%)	0.64
Total procedure duration, min	180 ± 35	176 ± 32	183 ± 36	0.34	179 ± 43	181 ± 40	0.11
Total fluoroscopy dose, μGycm^2^	2636 ± 540	2601 ± 480	2691 ± 550	1.14	2620 ± 532	2711 ± 534	0.92
Fluoroscopy duration, min	18 ± 8	17 ± 7	20 ± 9	1.8	19 ± 6	18 ± 9	0.87

### Procedural data

Acute PVI was achieved in all patients (*n* = 353, 100%). Procedural data of patients with a normal PV anatomy were compared to those patients with variant PV characteristics (Table 1). Although, procedural and fluoroscopy times were slightly longer in the variant PV-anatomy cohort of patients, this difference did not reach statistical relevance. Nadir temperatures and freezing times were similar between both groups (Table 2).

**Table 2:** Freezing time and nadir temperature for each PV depending on AF-recurrence. A *p*-value < 0.05 and bold letters state statistical significance. Continuous variables are shown as the mean ± SD. *AF* atrial fibrillation, *PV* pulmonary vein, *RSPV* right superior pulmonary vein, *RIPV* right inferior pulmonary vein, *LSPV* left superior pulmonary vein, *LIPV* left inferior pulmonary vein, *LCPV* left common pulmonary vein

	Freezing time, s			Nadir temperature, °C		
PV/group	Long-term AF-free survival	AF-recurrence	*p*-value	Long-term AF-free survival	AF-recurrence	*p*-value
RSPV	224 ± 28	210 ± 42	0.08	− 44 ± 7	− 41 ± 3	0.19
RIPV	230 ± 23	210 ± 34	0.67	− 45 ± 8	− 43 ± 4	0.39
LSPV	226 ± 25	200 ± 35	0.54	− 44 ± 6	− 47 ± 6	0.28
LIPV	229 ± 24	200 ± 25	0.32	− 40 ± 6	− 41 ± 3	0.09
LCPV	230 ± 23	238 ± 20	0.27	− 41 ± 5	− 42 ± 4	0.11

### Clinical outcome depending on PV anatomy

Two hundred twenty-three patients (63%) presented with a regular PV anatomy (2 right- and 2 left-sided PVs). A variant PV anatomy was present in 130 patients (37%) and distributed as follows: 27% (*n* = 95) left common ostium (LCPV), 5% (*n* = 16) right middle PV (RMPV), 5% (*n* = 19) right common ostium (RCPV). Further details are summarized in Table 3.

**Table 3:** Distribution of pulmonary vein anatomy types among patients with and without early (at 12 months) and late AF-recurrence (after one year). A *p*-value < 0.05 and bold letters state statistical significance. *PV* pulmonary vein, *AF* atrial fibrillation, *LCPV* left common pulmonary vein, *RCPV* right common pulmonary vein, *RMPV* right middle pulmonary vein

PV anatomy	Total study population (*n* = 353)	12-month AF-free survival (*n* = 285)	Early AF-recurrence (*n* = 68)	*p*-value	Long-term AF-free survival (*n* = 186)	Late AF-recurrence (*n* = 99)	*p*-value
Regular PV anatomy	223 (63%)	192 (67%)	31 (46%)	**< 0.001**	152 (82%)	40 (40%)	**< 0.001**
Variant PV anatomy	130 (37 %)	93 (33%)	37 (54%)	**< 0.001**	34 (18%)	59 (60%)	**< 0.001**
- LCPV	95 (27%)	75 (27%)	20 (29%)	0.61	34 (18%)	41 (41%)	**< 0.001**
- RCPV	16 (5%)	8 (3%)	8 (12%)	**< 0.001**	0 (0%)	8 (8%)	**< 0.001**
- RMPV	19 (5%)	10 (3%)	9 (13%)	**< 0.001**	0 (0%)	10 (10%)	**< 0.001**

#### 12-months’ follow up

Patients with AF-recurrence within 12 months (*n* = 68, 19%) significantly more often presented with a variant PV anatomy in comparison to patients with AF-free survival within this observation period (*n* = 37, 54% vs. *n* = 31, 46%, *p* < 0.001). Among those patients with early AF-recurrence, significantly more right-sided variant PVs were observed (*p* < 0.001). Details are presented in Table 3. Kaplan-Meier plot analyses demonstrated a significantly impaired 12-month AF-free survival rate in patients with right-sided variant PV characteristics (Log-rank *p* = 0.008).

#### 48-months’ follow up

One hundred sixty-seven patients (47%) have had AF-recurrence within the observation period of 48 months. Fifty-nine percent of them (*n* = 99) suffered from late AF-recurrence. Significantly more patients with variant PV characteristics (*n* = 59, 45%) presented with late AF-recurrence compared to those with a normal PV anatomy (*n* = 40, 18%) (*p* < 0.001) (Table 3).

**Fig. 2:**
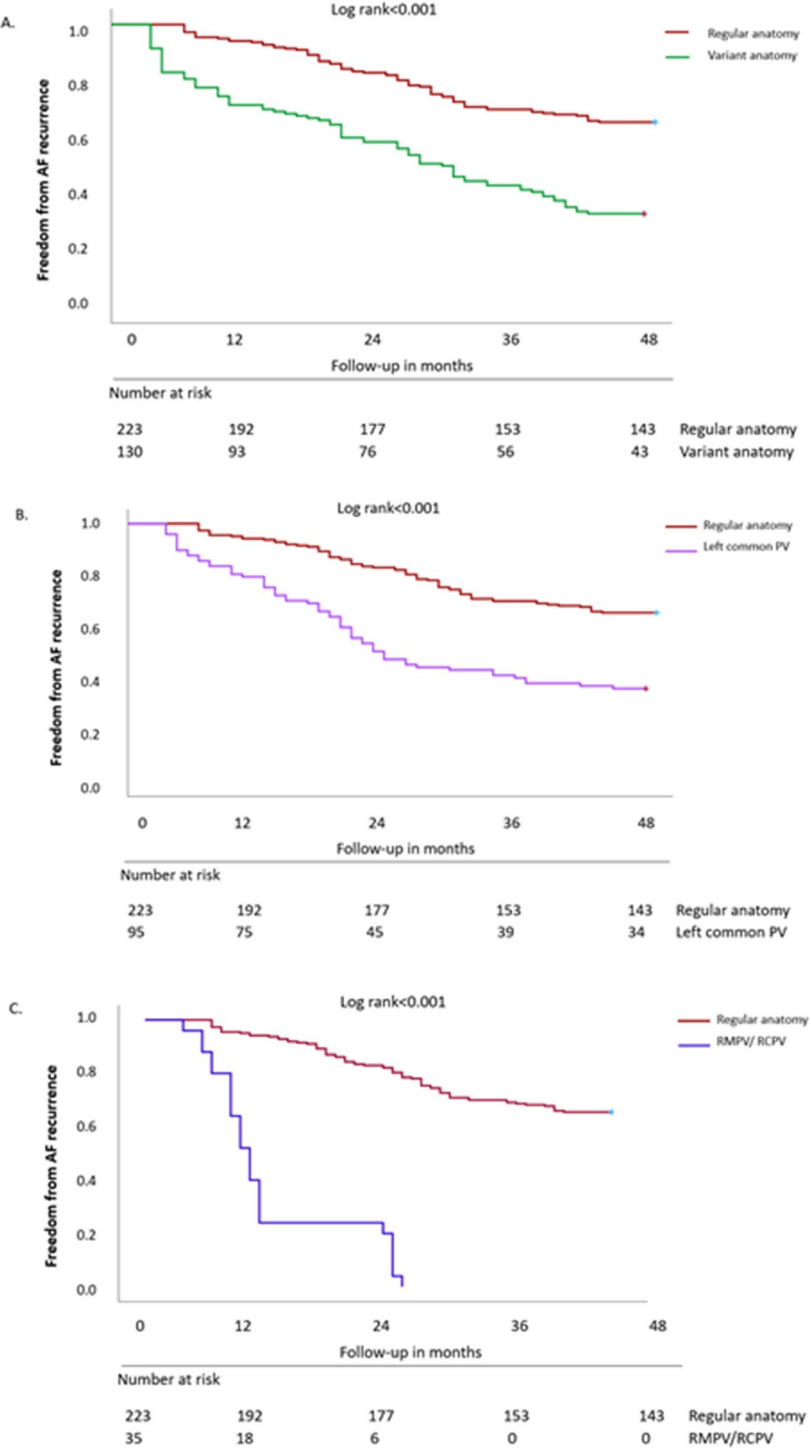
Kaplan–Meier plot on freedom from AF-recurrence. Comparison of regular (red) PV anatomy with **A** all types of variant PV anatomy (green); **B** LCPV (magenta); **C** right-sided PVs (blue). A *p*-value ≤ 0.05 indicates statistical significance. AF, atrial fibrillation; PV, pulmonary vein; LCPV, left common pulmonary vein; RCPV, right common pulmonary vein; RMPV, right middle pulmonary vein

**Fig. 3:**
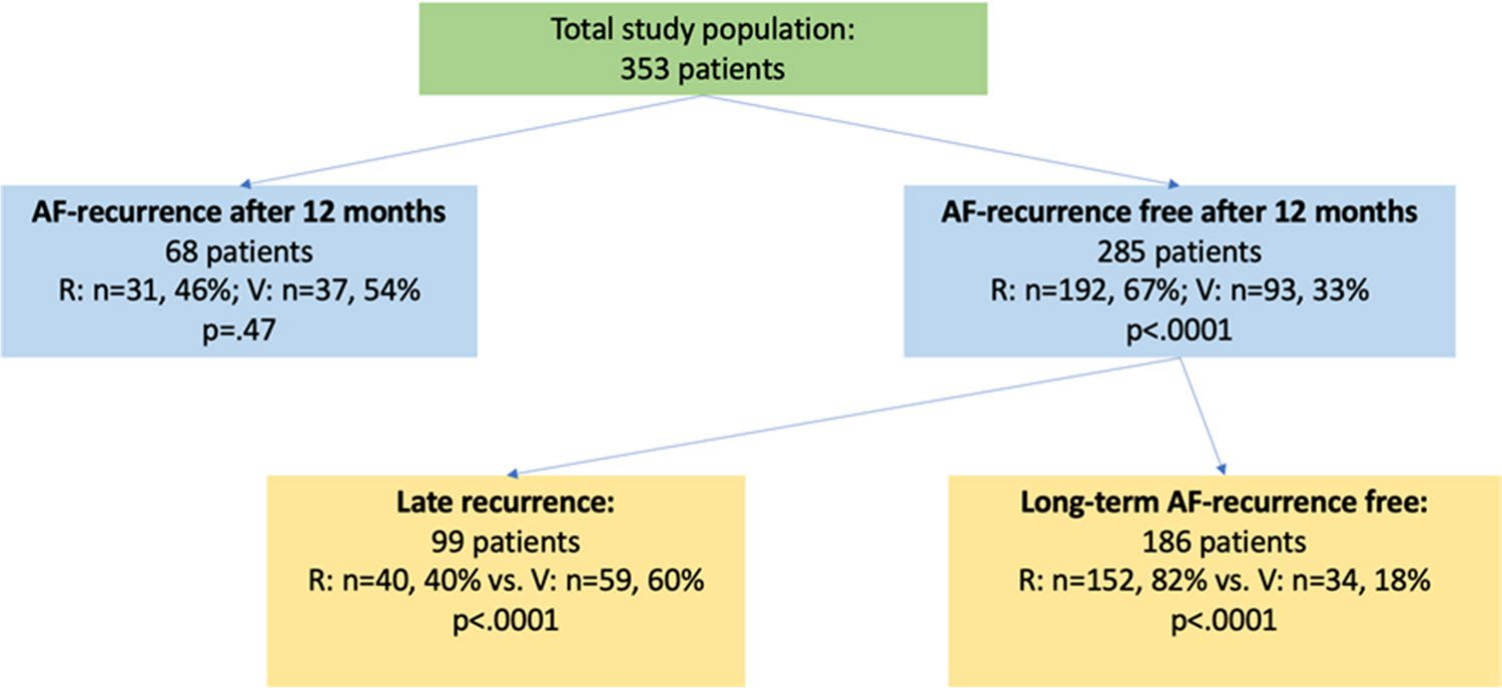
Schematic figure showing the study cohort and subgroups depending on AF-recurrence and distribution of regular and variant PV-anatomy among these groups. A *p*-value ≤ 0.05 indicates statistical significance. AF, atrial fibrillation; R, regular pulmonary vein anatomy; V, variant pulmonary vein anatomy; *n*, number of patients

No patient was lost to follow-up. Kaplan-Meier plot survival analyses demonstrate a significantly higher probability of AF-recurrence among patients with variant PV characteristics. Both, patients with a LCPV (Log-rank *p* < 0.001) and those with right variant PVs (Log-rank *p* < 0.001), present with a significantly worse long-term outcome following CBA. Details on patients’ AF-free survival are summarized in Figs. 2 and 3.

#### Clinical outcome depending on CSA

Concerning CSA dimensions, patients with AF-recurrence within the observation period of 48 months presented with significantly enlarged LSPVs and right-sided PVs as well as significantly greater dimensions of LCPVs. Details are gathered in Table 4.

**Table 4:** Impact of PV-CSA on AF-recurrence among patients with and without early (at 12 months) and late AF-recurrence (after one year). *PV* pulmonary vein, *CSA* cross-sectional area, *RSPV* right superior pulmonary vein, *RIPV* right inferior pulmonary vein, *LSPV* left superior pulmonary vein, *LIPV* left inferior pulmonary vein, *LCPV* left common pulmonary vein, *RCPV* right common pulmonary vein, *RMPV* right middle pulmonary vein. A *p*-value < 0.05 and bold letters state statistical significance

PV/CSA, mm^2^	Early AF-recurrence	12-month AF-free survival	*p*-value	Late AF-recurrence	Long-term AF-free survival	*p*-value
RSPV	260.0 ± 76.0	259.1 ± 66.7	0.45	263.1 ± 78.2	257.2 ± 75.3	**0.03**
RIPV	250.0 ± 93.3	234.8 ± 69.6	0.09	254.4 ± 96.3	248.7 ± 90.0	**0.04**
LSPV	234.8 ± 69.6	218.0±70.7	0.45	235.5 ± 67.3	226.8 ± 65.3	**0.01**
LIPV	163.0 ± 58.8	159.0 ± 44.7	0.33	162.2 ± 58.0	161.2 ± 59.1	0.41
LCPV	355.6 ± 60.0	311.0 ± 60.0	0.07	348.7 ± 59.0	322.7 ± 34.2	**< 0.001**
RCPV	490.3 ± 89.2	487.5 ± 79.3	0.91	508.6 ± 84.3	*	*
RMPV	64.2 ± 18.5	63.1 ± 15.4	0.89	71 ± 13.2	*	*

*All patients with right-sided PVs suffered from late AF-recurrence

#### Multivariate analysis

Multivariate Cox Regression model calculation revealed the presence of right-sided PVs and a LCPV as independent predictors for AF-recurrence (Table 5).

**Table 5:** Multivariate analysis of AF-recurrence within the observation period of 48 months: hazard ratio (95% confidence interval). *A p*-value < 0.05 and bold letters state statistical significance. *DM* diabetes mellitus, *CAD* coronary artery disease, *AADs* antiarrhythmic drugs, *LCPV* left common pulmonary vein, *LA* left atrium, *PV* pulmonary vein

	Hazard ratio, 95.0% CI	*p*-value
Baseline parameter		
Sex (male)	1.6 (0.9–2.63)	0.47
Smoking	0.99 (0.54–2.0)	0.91
Arterial hypertension	0.9 (0.72–1.15)	0.56
DM	0.49 (0.9–1.9)	0.88
CAD	0.40 (0.09–1.9)	0.63
LA > 40 mm	2.3 (0.76–3.03)	0.78
AADs	0.87 (0.43–2.6)	0.99
Anatomy		
LCPV	2.9 (1.4–5.8)	**0.03**
Right-variant PVs	1.9 (1.73–3.9)	**0.004**

### Complications

One patient (< 1%) developed a pericardial effusion after the procedure, and 3 patients (1%) had vascular groin complications (pseudoaneurysm of the femoral vein) not requiring intervention. No further postprocedural complications were observed.

## Discussion

This study has two major findings: First, variant PV anatomy seems to be predictive for long-term AF-recurrence in PAF. LCPVs and right variant PVs were revealed as independent predictors for AF-recurrence following CB-guided PVI. Second, a correlation between AF-recurrence and an enlarged CSA was observed for LSPVs and right-sided PVs.

### Variant pulmonary vein anatomy

In our previous study comparing two single-shot devices, patients with a variant PV anatomy presented with a significantly higher 12-month AF-recurrence rate (69%) compared to patients with a regular PV anatomy (44%) (*p* < 0.001) [8]. In our present study, we analysed patients’ 48-month long-term arrhythmia outcome. In line with the previously published study [8], variant PV characteristics were significantly more often observed in the AF-cohort compared to the No-AF group of patients (Table 3; Fig. 2). In detail, significantly more LCPVs, RCPVs and RMPVs were found in the AF-cohort of patients (*p* < 0.001) (Table 3; Fig. 2). The presence of variant PV characteristics, a right variant PV anatomy and a LCPV were revealed as independent predictors for AF-recurrence (Table 5).

Smaller CBA studies comparing AF-free survival rates depending on the presence of a LCPV reported on similar results [9, 10]. But compared to prior publications, our present study is the first to present 48 month long-term outcome data after single-shot guided CBA for PAF in a large cohort of patients depending on PV anatomy. Çöteli et al. compared AF-freedom after CBA and RFA depending on the presence of a LCPV in patients with PAF and PERS AF [11]. Mid-term AF/AT-recurrence-free survival between CBA and RFA did not differ significantly (*p* = 0.235). Similar results were presented by Khoueiry et al. comparing 15-month outcome data after CBA and RFA among patients with PAF and PERS AF depending on variant PV anatomy characteristics [12]. These results may be caused by the analysis of PAF and PERS AF as in our previous study, significant differences were observed between PAF and PERS AF. Especially in PAF, variant PV characteristics had a significant impact on AF-free survival whereas PV characteristics seem to play a minor role in PERS AF most likely due to advanced stages of atrial fibrosis and scar formation [8]. Moreover, different classifications and definitions for the left common ostium used in prior studies may in part explain discrepant results, too [11–13].

The incidence of a RMPV among AF-patients is being reported to be 20% [12]. In our study, 13% of all patients with AF-recurrence had a RMPV (Table 3). A meta-analysis of Cheruiyot et al., including 12 CBA and RFA studies, demonstrated a significant association between the presence of a RMPV and AF-recurrence (OR = 1.85, 95% CI 1.26–2.72, *p* = 0.002) [14]. The authors suggested that technical difficulties in reaching a small PV may be the reason for AF-recurrence in these patients. Additionally, they proposed that small PVs can be easily overseen during ablation [14].

These results match our findings, as patients with right-variant PVs presented with the poorest outcome (Fig. 2). The data given above suggests that a more complex understanding of PV characteristics is strongly required to improve patients’ outcome.

### Cross-sectional area of the pulmonary vein

The crucial moment in achieving acute PVI is not just the complete vein occlusion but also a wide antral ablation. It is well-recognized that antral positioning is more efficient in terms of long-term results [15]. It could be explained by involving more arrhythmogenic aerials of PV-LA connection sometimes also including the posterior wall of the LA. A wide antral ablation became more feasible after introducing the 2nd-generation cryoballoon. It allows for a shorter procedure duration and enables proper positioning in challenging PV anatomy [16, 17]. Although complete occlusion was routinely confirmed angiographically for each PV, we revealed a significant correlation between enlarged right-sided PVs as well as LSPVs and 48-month AF-recurrence rates (Table 4). These results act in concert with our previous study, analysing patients’ 12-month outcome data [8]. Another study by Güler et al. revealed the size of the RSPV as independent predictor for AF-recurrence [18]. This supports the hypothesis that PV anatomy seems to be an important issue [19]. Dilated PV orifices may lead to structural and electrical remodeling processes of the PVs that trigger AF recurrence. However, Bose et al. showed that PV-dimensions do not have a statistically significant impact on AF freedom [20]. These differences may be caused by procedural differences.

Further studies are warranted, e.g., comparing the impact of different ablation strategies (CBA vs. RFCA) on the outcome following catheter ablation for PAF in patients with variant PV characteristics.

## Limitations

Since the size of PVs is not constant and changing throughout the cardiac cycle and depending on hydration status and left atrial geometry, it may have an influence on the measurements done during preprocedural imaging. For each PV, The CSOA of the PVs was calculated using the following equation *p* × (1/2 × *D*1 × *D*2) allowing for an approximate, not an exact determination of the PV ostium dimension. The stable size of the 28-mm CB could have led to more distal ablation lesions in enlarged PVs. Thus, we cannot exclude an influence of rather distal ablation lesion in enlarged PVs on patients’ AF-free survival. The subgroup of patients with right variant PV characteristics is quite small. Further studies are warranted to confirm our initial observations.

## Conclusion

Variant PV anatomy predicted 48-month long-term AF-recurrence following single-shot–guided CBA. Patients with right-variant PVs presented with the worst outcome in terms of arrhythmia recurrence. A correlation between the CSA of right-sided PVs as well as LSPVs and AF-recurrence was demonstrated.
